# Hepatitis C virus genotype analysis in patients with chronic hepatitis in North Eastern Bulgaria

**DOI:** 10.1080/21556660.2019.1654484

**Published:** 2019-08-08

**Authors:** Zarina Brady, Zhivka Stoykova

**Affiliations:** a Medical University of Varna, Varna, Bulgaria;; b Department of Microbiology and Virology, Medical Faculty, Medical University of Varna, Varna, Bulgaria;; c Laboratory of Clinical Virology, University Hospital “St. Marina”, Varna, Bulgaria

**Keywords:** Hepatitis C virus, HCV genotype, HCV distribution, HCV prevalence, chronic liver disease

## Abstract

**Background:** The main objective of this study was to analyse the spread of hepatitis C virus (HCV) genotype in patients with chronic liver disease; commenting on the molecular characterization of HCV and gender and age in Varna, Bulgaria. Across Europe and the world, HCV is a significant economic concern and public health crisis. Defined by genotype variations, HCV is the leading cause of chronic liver disease, liver related morbidity, and mortality worldwide. Active examination for asymptomatic patients is essential, initiating early treatment aimed at the specific HCV genotype, effective outcomes, and reducing transmission and mortality in Bulgaria.

**Methods and materials:** Nucleic acid extraction and amplification were performed with commercially available test kits on 115 patients blood samples collected from March 2018 to October 2018. Male (*n* = 58) (50.43%, 95% CI = 41.29%–59.57%) and female (*n* = 57) (49.57%, 95% CI = 41.29%–59.57%) samples were equally distributed (mean age = 51.4 years; SD = ±16.5 years; range = 17–87 years old).

**Results:** Genotype 1b predominated (73%, 95% CI = 64.89%–81.11%), followed by high prevalence of 1a (13.9%, 95% CI = 7.58%–20.22%) and 3 genotypes (11.3%, 95% CI = 5.51%–17.09%). Genotypes 2 and 4 were equally the least prevalent (0.9%, 95% CI = −0.83%–2.63%). In genotype 1b, 60.7% were women and 39.3% were men; in genotype 1a, 25% were women and 75% were men; and in genotype 3, only 7.7% were women and 92.3% were men. Males were most prevalent in genotypes 1a (75%) and 3 (92.3%), while women were most prevalent in genotype 1b (60.7%).

**Conclusions:** HCV genotype lb is the predominant variant within the epidemiological pattern of HCV genotypes in patients with chronic liver diseases in North Eastern Bulgaria.

## Introduction

With Hepatitis C virus (HCV) being a leading burden to health globally, it is the number one cause of chronic liver disease, with serious complications such as cirrhosis, fibrosis, and hepatocellular carcinoma of the liver^1–3^. Studies note Eastern Europe and Eastern Mediterranean WHO regions to have the highest prevalence statistics, 1.5% and 2.3%, respectively^4^.

The prevalence of HCV in the Bulgarian population is 1.1%–1.5%^5^^,^^6^, with anti-HCV prevalence of 1.3%, and the high-risk group with the highest prevalence rate being people between 20–34 years old^7^.

Before 1992 literature lacked consistent HCV genotypes reported in Bulgaria, divided by geographic location, as blood and product screening began after this time^5^. The first reported HCV genotype distribution in Bulgaria, by Andonov *et al*.^8^ in 1996, noted HCV genotype lb to be the predominant variant within 117 Bulgarian patients, corresponding with our results.

A recent meta-analysis in 2016 states that there is no available data about HCV genotypes that are circulating in Bulgaria^9^.

This study investigated circulating HCV genotypes in 115 patients from NE Bulgaria, presenting with chronic liver disease, >6 months duration (anti HCV/HCV-RNA positive results), defining the molecular characterization according to the age and gender.

Seven genotypes are documented on the basis of phylogenetic and sequence analyses of whole viral genomes, genotype 1a + 1b, 2, 3, 4, 5, 6, and 7^10–12^. HCV strains display a wide genetic diversity, differing at ∼35% of nucleotide sites. Each genotype can be additionally classified into 67 confirmed and 21 provisional sub-types, with strains of the same family differing by <15% of nucleotide sites^11^. However, certain genotypes seem to be confined to a specific region of the world. Genotype la predominates in North America^13^^,^^14^, whereas genotype lb, which is more often associated with aggressive liver disease, is found more frequently in Western Europe and Japan. Genotype 2 is less common in Europe than in Asian countries such as China, Japan, and Taiwan^15^. Genotype 3 is often found in the UK and Thailand, while genotype 4 is associated with the Middle East and Central Africa^16–18^. The distribution of genotype 5 has been confined thus far mainly to South Africa^19^, and that of genotype 6 restricted to Hong Kong^20^. Genotype 7 has been reported in central Africa, isolated from patients in the Democratic Republic of Congo^11^^,^^12^.

Since 2018, patients with HCV infection can be treated with a ribavirin-free direct-acting antiviral drug, for example Mavyret (glecaprevir and pibrentasvir), Sofosbuvir, Velpatasvir or Voxilaprevir^2^. Mavyret is suitable for patients with HCV genotypes 1–6, given once daily over an 8-week period, and has shown extremely successful eradication rates of 98%^21^.

## Methods

A total of 115 patients with chronic hepatitis, 58 males (50.43%, 95% CI = 41.29%–59.57%) and 57 females (49.57%, 95% CI = 41.29%–59.57%) (mean age of 51.4 years, SD = ±16.5 years, and range = 17–87 years old) from Varna, Ruse, Silistra, Dobrich, and Razgrad, were investigated from March to October 2018, at the Laboratory of Clinical Virology, University Hospital “St. Marina”, Varna, Bulgaria. Inclusion criteria included all patients that were anti HCV positive in ELISA screening. Each patient signed informed consent documents and were investigated by their will in order to start examinations and ultimately the correct diagnosis and treatment.

The patient cohort were anti HCV positive in screening or tested on demand due to elevated liver enzymes. HCV viral load and HCV genotype was determined for the very first time in order to start treatment if necessary. Staging of patient liver disease and treatment was further evaluated in the Gastroenterology Department after the viral load and HCV genotype results. Regrettably, data was not collected on patient HIV status; however, patients did not belong to at risk groups.

Nucleic acid extraction, amplification, and HCV genotyping were performed with commercially available test kits according to the manufacturer’s recommendations. Magrev Viral DNA/RNA Extraction Kit (Anatolia Geneworks, Istanbul, Turkey) was used for the manual extraction of HCV-RNA from 500 µl plasma samples in a 60 µl elution volume. Anatolia Bosphore HCV Genotyping Kit v3 (Anatolia Tani ve Biyoteknoloji A.S., Istanbul) was used to characterize the HCV genotype, with sensitivity of 100 IU/ml, a region within the NS5B was amplified and fluorescence detection is accomplished using the FAM and HEX filters. The test identifies the six major and most common HCV genotypes (1, 1a, 1b, 2, 3, 4, 5, 6). Amplification was performed with PCR instrument Quant Studio DX.

The proportion of the individuals with a particular genotype and their corresponding confidence intervals were calculated using McCallum Layton calculators. The mean age of the participants and standard deviation of it was calculated using a Microsoft Excel 97-2003 worksheet (.xls). No additional statistical tests were carried out due to limited patient information.

## Results

**Figure 1.**
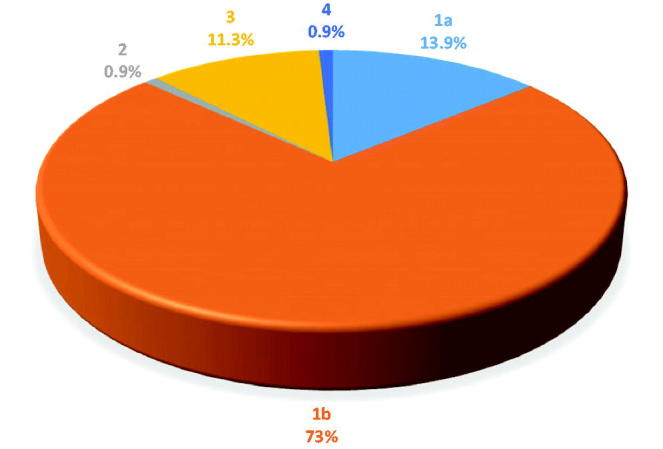
Distribution of HCV genotypes in North Eastern Bulgaria.

Our study demonstrated the HCV serology status of the patients, age, and gender. The predominance of genotype 1b (73%, 95% CI = 64.89%–81.11%), followed by high prevalence of 1a (13.9%, 95% CI = 7.58%–20.22%) and 3 genotypes (11.3%, 95% CI = 5.51%–17.09%). Genotypes 2 and 4 were the least prevalent, seen in only 0.9% (95% CI = −0.83% to 2.63%) of the studied population each (Figure 1).

**Figure 2.**
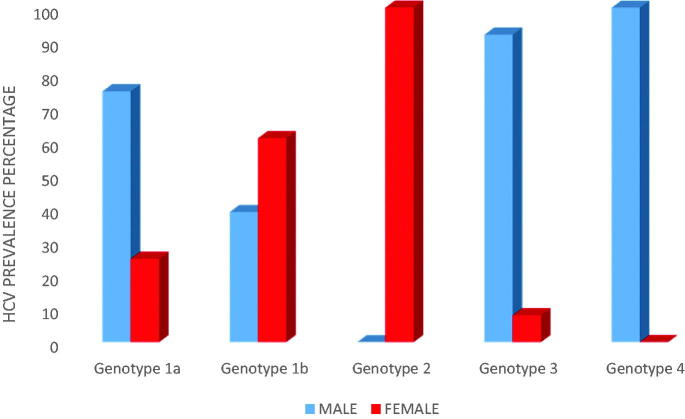
Prevalence of HCV genotypes by gender in North Eastern Bulgaria.

Out of the 1a genotype the most prevalent were found to be men (75%). Out of the 1b genotype most prevalent were found to be women (60.7%). Out of the 3 genotype the most prevalent were found to be men (92.3%) (Figure 2). The viral load varied from 488–6,971,487 IU/ml (average = 565,201 IU/ml) determined with a commercially available test kit from Geneworks, Anatolia, Bosphore (Table 1).

**Table 1.** Comparison of the HCV genotyping and viral load.

HCV genotype	Number of patients (*n* = 115)	Average viral load (IU/ml)
1a	16	446,007
1b	84	443,365
2	1	55,643
3	13	1,629,623
4	1	50,678

**Figure 3.**
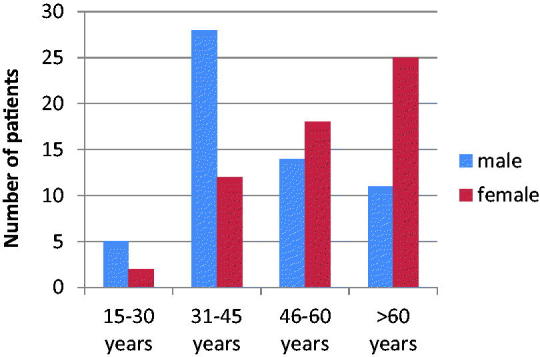
Age and gender distribution in HCV infected patients.

The highest number of HCV infected men were between the age of 31–45 years (48.3%; *n* = 28), while the highest number of HCV infected women were over 60 years (43.9%; *n* = 25) (Figure 3).

## Discussion

HCV screening massively differs from country to country across Europe, disputing collation methods, reporting, and testing practices^22^. Bad practices in the Bulgarian healthcare system are the lack of any reimbursed HCV screenings or early diagnostic programs and treatment of small group of patients responding to the strict selection criteria. The European Center for Disease Prevention and Control noted 27 countries to have 38 different systems, with six of the countries having more than one system^23^, thus a high level of awareness should be used when examining epidemiology from various countries.

European studies reveal a low variability between the spread of genotypes in Central, Eastern, and Western Europe. Genotype 1 had a low variability in Central and Eastern Europe, 70% and 68.1%, respectively, while comparing the three areas genotype 2 had the high prevalence amongst Western European countries (8.9%). Western and Eastern Europe had the highest rates of genotype 3 (29% and 26.6%), and Western and Central Europe areas had a genotype 4 spread of 4.9% and 5.8%, respectively^9^.

In Bulgaria in 2014 an estimated 18,000 patients were diagnosed with chronic HCV infections, with 1,200 newly-diagnosed annually over the last 15 years. According to the Bulgarian National Health Insurance Fund, 720 patients were treated, 70 patients with triple therapies, 180 patients with direct acting antivirals therapy, and 470 patients with Pegylated interferon therapy in 2015^6^.

A study quotes 1.1% of the Bulgarian population to have HCV, with a massive 87% leading to chronic HCV infection^24^^,^^25^. Similarly, a Bulgarian population consensus published in 2012 revealed an anti-HCV prevalence of 1.5%, with 95,000 viraemic cases, and RNA‐positive infections^26^.

Maaroufi *et al*.^6^ addressed the requirement for country‐level assessments of HCV‐infected populations. From 1991–2013 genotypes were established as an overall genotype 1a (20%), 1b (62%), 2 (1%), 3 (14%), and 4 (1%)^6^. In another study performed among 117 patients, the most prevalent genotype is reported to be 1b (72.3%)^8^. Similarly, out of our 115 patients tested, genotype 1b (73%) was the most prevalent, followed by 1a (13.9%), 3 (11.3%), with the least prevalent being genotypes 2 (0.9%) and 4 (0.9%). The two patients with determined genotype 2 and 4 have never travelled abroad.

A study by Ganova-Raeva *et al*.^27^ noted HCV transmission of subtype 1a to be most prevalent (54%), followed by 1b (20.8%), 2a (1.4%), 3a (22.3%), and 4a (1.4%), in high risk drug addicted population groups of HIV/HCV coinfections in Western Bulgaria, Sofia, Plovdiv, and Peshtera^27^. However, this study includes patients from another region, whereas our study describes North Eastern Bulgaria. Additionally, we do not have data on risk factors, which is the most probable explanation for the differences between the studies.

There are several limitations to this study. One limitation being the patient sample size, a larger investigation at a national level could be undertaken to analyse the prevalence of HCV as a whole of the Bulgarian population. Additionally, the authors did not note the exact origin of each patient, or how they were infected.

## Conclusion

In conclusion periodic investigation into the molecular identification of such rising sub-types and strains in Bulgaria may be appropriate in examining methods of prevention and routes of transmission of hepatitis. HCV genotype lb, followed by the high prevalence of 1a and 3 genotypes are the predominant variants within the epidemiological pattern of HCV genotypes in patients with chronic liver diseases in North Eastern Bulgaria. We highlight the need for greater observation of HCV genotypes at a national level, in order to identify asymptotic patients and thereby decrease early mortality rates, limiting spread and excessive morbidity rates. Robust national efforts, strict rules on larger data collection, examining high risk patients, and a central register may be the key in limiting HCV spread at European and other continental levels.

## Transparency

### Declaration of financial/other relationships

There are no relationships to be declared. JDA peer reviewers on this manuscript have no relevant financial or other relationships to disclose.
